# Early syphilis: risk factors and clinical manifestations focusing on HIV-positive patients

**DOI:** 10.1186/s12879-019-4269-8

**Published:** 2019-08-16

**Authors:** Maider Arando, Candela Fernandez-Naval, Miriam Mota-Foix, Desi Martinez, Pere Armengol, Maria Jesús Barberá, Juliana Esperalba, Martí Vall-Mayans

**Affiliations:** 1 STI Unit Vall d’Hebron-Drassanes, Infectious Diseases Department, Hospital Vall d’Hebron, Av Drassanes 17-21 STI Unit Vall d’Hebron-Drassanes, Barcelona, Spain; 2 Medicine Department, Autonomous University of Barcelona, Barcelona, Spain; 3 Vall d’Hebron Institute of Research, Barcelona, Spain; 4 Genetics and Microbiology Department, Autonomous University of Barcelona, Barcelona, Spain; 5 Statistics and Bioinformatics Unit, Vall d’Hebron Institute of Research, Barcelona, Spain; 6 Microbiology Department, Hospital Vall d’Hebron, Barcelona, Spain

**Keywords:** Syphilis, Men who have sex with men, Condomless anal sex, HIV

## Abstract

**Background:**

Since 2000, substantial increases in syphilis in men who have sex with men (MSM) have been reported in many cities. Condomless anal sex (CAS) is one of the factors, along with drugs for sex and sex in group. This study identified factors and clinical manifestations as well as *Treponema pallidum (T.pallidum)* strains that could be related to early syphilis in Barcelona.

**Methods:**

This prospective study was conducted in a sexually transmitted infections unit in 2015. Epidemiological, behavioral, clinical and microbiological variables were collected in a structured form. Univariate and multivariate statistical analyses were performed focusing on HIV-positive patients.

**Results:**

Overall, 274 cases were classified as having early syphilis (27.5% primary, 51.3% secondary, and 21.2% early latent syphilis). In all, 94% of participants were MSM and 36.3% were HIV-positive. The median number of sexual contacts in the last 12 months was 10; 72.5% practiced CAS, 50.6% had sex in group, and 54.7% consumed drugs. HIV-positive cases had more anonymous sex contacts (*p* = 0.041), CAS (*p* = 0.002), sex in group (*p* < 0.001) and drugs for sex (*p* < 0.001). In the multivariate analysis, previous syphilis (adjusted odds ratio [aOR] 4.81 [2.88–8.15]), previous *Neisseria gonorrhoeae* infection (aOR 3.8 [2.28–6.43]), and serosorting (aOR 20.4 [7.99–60.96]) were associated with having syphilis. Clinically, multiple chancres were present in 31% of cases with no differences on serostatus, but anal chancre was most common in HIV-positive patients (*p* = 0.049). Molecular typing did not conclusively explain clinical presentation in relation to specific *T.pallidum* strains.

**Conclusion:**

Control of syphilis remains a challenge. Similar to prior studies, HIV-positive patients were found to engage more often in sexual behaviors associated with syphilis than HIV-negative patients. Clinical manifestations were rather similar in both groups, although anal chancre was most common in HIV-positive patients. Various strain types of syphilis were found, but no clinical associations were identified.

## Background

Since 2000, substantial increases in cases of early syphilis have been reported in metropolitan areas of Western countries. These increases have been described particularly among men who have sex with men (MSM). In Europe, there were 28,701 cases of early syphilis reported in 2015, yielding a rate of 6.0 per 100,000 inhabitants [1]. Similarly, an increase has been described in our setting in Catalonia (Spain): in 2014, the rate of early syphilis was 12.4 per 100,000 inhabitants, a 231% increase over the figures for 2005 observed mainly in MSM [2].

One of the most important factors affecting syphilis transmission is the practice of condomless anal sex (CAS) [3]. Other factors, such as drug consumption, internet use for sexual contacts, and sex in group have also been reported [4]. In some studies, these risk factors are directly related to syphilis, whereas, other studies have found, indirect links with CAS [5, 6]. HIV coinfection has been demonstrated to be strongly associated with syphilis [3, 7].Clinically, some differences have been described between HIV-positive and HIV-negative patients, for instance, in atypical manifestations, such as multiple chancres in primary syphilis [8] or the persistence of chancres in secondary syphilis [9] in HIV-positive patients.

Molecular typing of *Treponema pallidum* subspecies *pallidum* (*T. pallidum)* has been used for different purposes [10–12]. Epidemiologically, use of the technique has identified a wide distribution of strain types depending on geographic location, with 14d/g being the most common in Europe [13, 14]. Some strains have been associated with clinical outcomes such as neurosyphilis in rabbits, although this has not been shown in humans [10].

The aim of this study was to provide updated information on syphilis in Barcelona by identifying factors associated with early syphilis and occurrence of these factors, focusing on HIV-positive patients. An additional aim was to describe the clinical characteristics of syphilis, exploring the role of specific strain types of *T. pallidum*. Altogether, the study was intended to gain an overview of the syphilis epidemic that affects MSM in particular.

## Methods

This prospective study was conducted in the Sexually Transmitted Infections Unit Vall d’Hebron-Drassanes in Barcelona, Spain (STIUVD). The STIUVD is the main referral sexually transmitted infections (STIs) in Catalonia and is located in the downtown area of Barcelona. It provides care to patients for a total of 25000 visits per year (60% MSM) and reported around 50% of all syphilis cases seen in Barcelona during the study period.

All patients aged 18 years or older with early syphilis who came between 15 January and 15 October 15, 2015 were eligible to participate in the study. Syphilis diagnosis and management were based on the European syphilis guidelines [15]. Primary syphilis was defined as a typical ulcer (chancre) and/or a positive test using dark-field microscopy or as polymerase chain reaction (PCR) detection of *T. pallidum* and/or a positive serological test for syphilis. Secondary syphilis was defined based on typical clinical symptoms with positive treponemal and non-treponemal tests. Early latent syphilis was defined as positive serological treponemal and non-treponemal tests with no clinical evidence of infection, with a previous negative syphilis serology, or a four-fold increase in the titer of a non-treponemal test (i.e. two dilutions) within the past 12 months. Cured syphilis was defined as a four-fold decline in the titer of a non-treponemal test within 1 year of treatment.

### Behavioral variables

Participants completed a self-administered questionnaire of demographic and sexual behavior information from the previous 12 months. Variables included how they met their partners and practices such as sex in group, drugs for sex, use of condoms, serosorting (sex between partners with same HIV status) and seropositioning (adapting sexual practices according to one’s HIV status). The self-administered questionnaire was based on the European MSM Internet Survey (EMIS) [16], which had been previously modified, piloted, and revised. An adapted version of this questionnaire was used for heterosexual participants. Drugs for sex was defined as substance use 2 h before or during intercourse; chemsex was defined as the use of crystal methamphetamine, gammahydroxybutrate (GHB), and/or mephedrone before or during sexual sessions; and poly drug use was defined as the consumption of three or more drugs, excluding alcohol or cannabis. CAS was defined as the absence of systematic use of a condom, even if used occasionally. Clinical and microbiological variables were completed by the attending physicians. Information on pre-exposure prophylaxis against (PrEP) HIV was not collected because it was not readily available at that time.

If patients had more than 1 episode of syphilis during the study period, the epidemiological and behavioral data were analyzed only once. To determine the risk factors for the acquisition of syphilis according to HIV status, HIV-positive patients were defined as patients who knew that they were HIV-positive at syphilis diagnosis, excluding patients whose diagnoses of HIV and syphilis infection coincided. However, in the clinical analysis, the latter (patients with coincident diagnoses) were included in the HIV coinfection group analysis because the clinical manifestations and course of syphilis could be influenced by HIV.

### Microbiological tests

All patients were tested for syphilis following a reverse algorithm sequence screening algorithm. Initial screening consisted of a specific test for antibodies against *T. pallidum* with any positive results a subsequently confirmed by a non-treponemal test and another treponemal test. The tests used were the treponemal enzyme immunoassay (EIA, Siemens Healthcare Diagnostics, Germany), rapid plasma regain (RPR) test (Biokit, Spain), *T. pallidum* particle hemagglutination assay (TPHA, Biokit, Spain), and indirect immunofluorescence assay (FTA-abs, BioMérieux, France). Direct diagnoses were made using dark-field microscopy or in-house PCR for *T. pallidum* [17]. A swab was taken in the case of ulcer, with a second swab taken if there were lesions in more than one site. For typing purposes an extra swab was taken. Treponemal DNA was detected by means of a diagnostic real-time PCR (qPCR) and molecular typing was performed by the Enhanced CDC Typing, analyzing the *arp*, *tpr* and *tp0548* genes. This typing is based on three regions of *T. pallidum*-2 first described in 1998 by the Centers for Disease Control and Prevention (CDC) [18] and extended with *tp058* in 2010 by Marra et al. [10] Participants were offered screening for *N. gonorrhoeae* (NG), *C. trachomatis* (CT) and HIV, as well as hepatitis C virus (HCV) in MSM.

### Statistical analysis

Categorical variables were expressed as percentages and comparisons were made using the chi-square test or Fisher’s exact test, as appropriate. The strength of the association was measured as the odds ratio (OR) with 95% confidence intervals (95% CI). Continuous variables were expressed as the mean (+/− SD) or median and interquartile range [IQR]. Comparisons were performed using a Student t test, Mann-Whitney U test, ANOVA, or Kruskal-Walls test with 95% CI if there were more than two groups. Variables identified as clinically significant at the univariate level (*p* < 0.01) were tested in a final regression model using the Akaike information criterion (AIC). The “R” program (R Foundation for Statistical Computing, Vienna, Austria), version 3.3.1, was used for all analyses.

Ethical approval was granted by the Hospital Vall d’Hebron Ethics Committee (PR(AG)297/2014) and all patients provided written informed consent obtained by the attending physician.

## Results

Among patients diagnosed with early syphilis during the study period, 33 patients declined to participate while another 3 were < 18 years old. In total, 270 patients with 274 episodes (4 patients had 2 episodes during this period) were included. In terms of episodes, 76 (27.7%) were primary syphilis, 140 (51.1%) were secondary syphilis and 58 (21.2%) were early latent syphilis. Most patients (257 [95%]) were MSM including bisexuals; there were 2 (0.75%) women and 11 (4.0%) heterosexual men. The median age was 36 [30–44] years, 60.5% were autochthonous, 65.0% had university-level education, 73.5% were employed, and 7.0% were students. A total of 95 (36.0%) patients knew they were HIV-positive at the time of syphilis diagnosis. Four patients had never undergone an HIV test (these patients were excluded from the comparative study). In HIV-positive patients, the median CD4 count was 653 cells/μl [517–810] and 85 (89.5%) were on antiretroviral therapy (ART). Among all patients, 42.8% (110) had a previous history of syphilis.

A total of 266 patients completed the questionnaire answering more than 85% of the questions (2 cases were excluded from the behavioral analysis due to incompleteness). Overall, in the past 12 months, the median number of contacts was 10 and CAS was practiced in 72.5% of cases. Half (50.6%) of them engaged in sex in group and 54.7% used drugs for sex- 25.0% practiced chemsex and 22.6% engaged in poly drug use. Two-thirds of patients searched for sexual partners through apps and 34.5% through the internet (Table 1).

**Table 1:** Risk factors related to early syphilis, overall and by HIV serostatus. Barcelona 2015

		HIV negative *N* = 167% 95%CI	HIV positive *N* = 95% 95%CI	ALL *N* = 266^a^ %	*p* Value	aOR (95% CI)
Sexual practices and condomless anal sex (CAS)	CAS	104	75	183	0.002	
		65.5	84.3	72.5		
		(57.4, 72.7)	(75.0, 91.1)			
	Receptive anal sex	107	77	185	0.011	
		67.3	82.8	72.3		
		(59.4, 74.5)	(73.5, 89.8)			
	Receptive CAS	40	46	88	0.005	
		39.6	62.2	49.7		
		(30.0, 49.8)	(50.1, 73.2)			
	Number of casual partners	*10*	*10*	*10*	0.041	
		[*4-5*]	*[5.75–32.8]*	*[4-20]*		
	Number of casual partners with anal sex	*5*	*6.5*	*6*	0.039	
		[*2-12*]	*[4–21.5]*	[*2-15*]		
	Number of casual partners with CAS	*1*	*3*	*1*	0.001	
		*[0–2.25]*	[*1-7*]	*[0-4]*		
Sexual risk behaviors	Serosorting	4	28	32	< 0.001	20.4
		3	43.1	16		[7.99–60.96]
		(0.8, 7.6)	(30.8, 55.9)			
	Seroposition	10	15	25	0.004	
		8.4	25.4	13.8		
		(4.1, 14.9)	(14.9, 38.4)			
	Contact through App	99	65	166	0.164	
		61	70.7	64.3		
		(53.1, 68.6)	(60.2, 79.7)			
	Contact through internet	55	34	89	0.729	
		34	37	34.5		
		(26.7, 41.8)	(27.1, 47.6)			
	Club sex	27	27	54	0.027	
		16.7	29.3	20.9		
		(11.2, 23.3)	(20.3, 39.7)			
	Sauna	33	17	50	0.841	
		20.4	18.5	19.5		
		(14.4, 27.4)	(11.1, 27.9)			
	Fisting	28	21	50	0.232	
		18.1	25.6	20.7		
		(12.3, 25.0)	(16.6, 36.4)			
	Sex in group	66	59	125	< 0.001	
		41.8	69.4	50.6		
		(34.0, 49.8)	(58.4, 78.9)			
	Toys	53	39	93	0.06	
		33.3	46.4	37.7		
		(26.0, 41.2)	(35.4, 57.6)			
	Drugs for sex	78	66	145	< 0.001	
		46.7	71	54.7		
		(38.9, 54.5)	(60.6, 79.9)			
	Cocaine	21	27	49	0.096	
		26.4	41.5	34		
		(17.5, 38.1)	(29.4, 54.4)			
	Mephedrone	4	12	16	0.024	
		5	18.5	11		
		(1.4, 12.6)	(9.9, 30.0)			
	GHB	22	35	58	0.003	
		28.2	53.8	40.3		
		(18.6, 39.5)	(41.0, 66.3)			
	Sildenafil	20	29	49	0.028	
		25.6	44.6	34		
		(16.4, 36.8)	(32.2, 57.4)			
	Crystal methamphetamine	9	19	28	0.015	
		11.5	29.2	19.5		
		(5.4, 20.7)	(18.6, 41.8)			
	Poly drug use	27	34	61	0.05	
		16	37	22.6		
		(10.9, 22.6)	(23.2, 46.2)			
	Chemsex	26	40	67	0.001	
		15.5	42	25		
		(10.4, 21.9)	(32.0, 52.6)			

+in italics: median with interquartile range in brackets

^a^four cases with HIV unknown serostatus

Clinically, in primary syphilis, genital chancre was the most common manifestation in 73.7% (56/76) of cases followed by anal chancre in 22.4% (17/76). Others locations (such as oral or urethral) were uncommon, less than 4% (3/76). All (except 2 cases) were confirmed by positive dark-field microscopy or PCR positive for *T. pallidum*. Oral chancre was found in 2 patients. Notably, 31.1% had more than one chancre and 2 patients had genital and anal chancres simultaneously. Coexistent chancres in secondary syphilis were observed in 38 (28.5%) of patients. The most frequent secondary lesions were syphilides in the genital area (70.7%) and skin rash (62.1%) affecting the soles (45.0%) more often than the palms of the hands (40.7%). The most common systemic manifestations were asthenia (27.1%), sore throat (22.1%), lymph node enlargement (10.7%), and malaise (10.7%) (Table 2).

**Table 2:** Clinical manifestation of early syphilis. Barcelona 2015

	HIV-negative	HIV-positive^b^	All	*P* Value
Primary syphilis	*N* = 59^a^ %	*N* = 17 %	*N* = 76 %	
Genital chancre	46	10	56	0.129
	78	58.8	73.7	
Anal chancre	10	7	17	0.049
	17	41.2	22.4	
Multiples chancres	17	6	23	0.553
	29.3	37.5	31.1	
Secondary syphilis	*N* = 75	*N* = 65	*N* = 140	
	%	%	%	
Skin eruption	51	36	87	0.174
	68.0	55.4	62.1	
Palm lesion	30	27	57	0.99
	40.0	41.5	40.7	
Sole lesion	36	27	63	0.551
	48.0	41.5	45	
Genital syphilides	54	45	99	0.863
	72.0	69.2	70.7	
Sore throat	16	15	31	0.965
	21.3	23.1	22.1	
Asthenia	18	20	38	0.479
	24.0	30.8	27.1	
Malaise	8	7	15	1
	10.7	10.8	10.7	
Lymph node enlargement	11	4	15	0.1
	14.7	6.15	10.7	

^a^including 3 other cases with oral and urethral chancres

^b^including patients diagnosed coinciding with syphilis infection

The median RPR titre at diagnosis was 1/32 [1/8; 1/64]. Among patients offered STI screening, 257 (94.0%) of them received NG and CT / *lymphogranuloma venereum* tests, with a prevalence of 9.3 and 16.0%, respectively. HIV was tested in 148 patients with 8.8% (13/148) being newly diagnosed while 5 (2.7%) of 189 patients tested positive for HCV (Table 3).

**Table 3:** Microbiological results (RPR and coinfection) of early syphilis. Barcelona 2015

	HIV negative	HIV positive^a^	All	*P* Value
	*N* = 168 61.3%	*N* = 106 38.3%	*N* = 274 100%	
RPR	*1/16*	*1/64*	*1/32*	< 0.001
	*[1/4–1/64]*	*[1/32–1/64]*	*[1/8;1/64]*	
STI coinfection^b^				
*Neisseria gonorrhoeae*	12	12	24	0.378
	7.69	11.9	9.3	
*Chlamydia trachomatis*	24	17	41	0.949
	15.4	16.8	15.9	
*Lymphogranuloma venereum*	4	6	10	0.267
	18.2	37.5	26.3	
*Hepatitis C virus*	3	2	5	0.637
	2.29	3.64	2.67	

^+^in italics: median with interquartile range in brackets

^a^including patients diagnosed coinciding with syphilis infection

^b^STI screening done in 257 patients for *N. gonorrhoeae* and C*. trachomatis/L. venerum* and 189 patients for *hepatitis C*

The majority of patients were treated with benzathine penicillin G 2.4 million units IM as a single dose (93.0%) and 14 (5.2%) were treated with oral doxycycline 100 mg twice daily for 14 days. About a year after treatment, a four-fold decrease in the non-treponemal test titre was observed in all but 14 patients (2 patients were reinfected, 1 had a serological failure and 11 were missing). Five patients became HIV-positive and 4 new cases of HCV occurred during the first year after syphilis diagnosis. Furthermore, 14 new syphilis reinfection cases occurred, 5 of them in HIV-positive patients.

Molecular typing was performed in 78 anogenital ulcers samples (16 samples could be typed only partially): 55 were from genital ulcers and 23 from anal ulcers. A high variety of strains was found making clinical associations unreliable. The strain type distribution (compared by proportions and graphics) between genital and anal chancre was similar, with 14d/g isolated most often in both (12.7% in genital chancre; 30.4% in anal chancre), followed by 14f/g (9.0% in genital chancre; 17.3% in anal chancre). In multiple chancres, 14d/g was also most common (20.0%). One patient with concomitant genital and anal chancres had different types in anal chancre (14f/g) compared with genital chancre (−p/g partial result).

### Comparative study by HIV status

All HIV-positive patients were MSM. Compared with HIV-negative patients, HIV-positive patients were older (39 [34–44] vs 34[29–43] years, *p* = 0.01), and were more likely to have had previous episodes of NG, CT, and syphilis, as well as HCV coinfection (Table 4). They also had more anonymous contacts in the past 12 months (87.0% vs 91%) with a median of 10 [5.75–32.8],3 [1-7]of which were CAS. There was no difference between the group in insertive anal sex; however, HIV-positive patients had more receptive anal sex (82.8% vs. 67.3%; *p* = 0.011) (Table 1). Concerning condom use for anal sex, HIV-positive patients were more likely to practice CAS (84.3%) than HIV-negative patients (65.5%; *p* = 0.002). Remarkably, 43.1% of HIV-positive patients practiced serosorting and 25.4% practiced seropositioning, while HIV-negative patients more frequently had CAS after having been tested for HIV (Table 1). Other risk factors were also more common in HIV-positive patients, such as having sex in group (69.4%), use of toys (46.4%), and drugs for sex (71.0%), mainly involving cocaine, GHB, mephedrone, sildenafil, and crystal methamphetamine. Poly drug use was reported by 34 HIV-positive patients (35.7%) and chemsex by 40 (42.1%). Among HIV-negative patients, 27 (16.1%) reported poly drug use (*p* = 0.05), and 26 (15.5%) reported chemsex (*p* = 0.001). Four patients, all HIV-positive, reported IV drug use. The final regression model showed that HIV-positive patients with syphilis were more likely to have previous syphilis (adjusted-OR, (ad-OR) 4.81 [2.88–8.15)] and previous NG infection (ad-OR 3.8 [2.28–6.43)]. Although mainly related with people with HIV, serosorting (ad-OR 20.4 [7.99–60.96)] was strongly associated with syphilis in HIV-positive patients.

**Table 4:** Previous STI related to early syphilis, overall and by HIV serostatus. Barcelona 2015

	HIV negative *N* = 167% (95% CI)	HIV positive *N* = 95% (95% CI)	All *N* = 266^a^ %	*p* Value	aOR (95% CI)
Previous STI					
Syphilis	42	68	110	< 0.001	4.81
	26.4	72.3	42.8		[2.88–8.15]
	(19.7, 33.9)	(62.1, 81.0)			
*N. Gonorrhoeae*	42	59	101	< 0.001	3.8
	26.8	66.3	40		[2.28–6.43]
	(20.0, 34.4)	(55.5, 75.9)			
*C. Trachomatis*	20	31	51	< 0.001	
	13.3	38.3	21.5		
	(8.3, 19.8)	(27.7, 49.7)			
HCV coinfection	3	13	16	< 0.001	
	1.8	13.7	6		
	(0.3, 5.1)	(7.5, 22.2)			

^a^four cases with HIV unknown serostatus

Clinically, in HIV-positive patients, secondary syphilis was the most common stage (61.9%). Primary syphilis was seen less often (15.2%), with a similar proportion to early latent syphilis (22.6%) compared with HIV-negative patients (early latent syphilis in 20.2%,secondary syphilis in 44.5%, primary in 35%; *p* = 0.002). After stratifying for previous syphilis as a possible confounding factor, the difference was still significant, secondary syphilis occurred more often in HIV-positive patients without a previous history of syphilis. Anal chancres were more common in HIV-positive patients than in HIV-negative patients (41.2% vs. 17.0%; *p* = 0.04). In secondary syphilis, there were no significant difference between these two groups (Table 2), even in terms of coexistent chancres. Median RPR titers were higher in HIV-positive patients at 1/64 [1/32–1/64] than in HIV-negative patients at 1/16 [1/4–1/64] *p* < 0.001 (Table 3). RPR titres stratified by previous history of syphilis showed that this difference persisted among patients without a previous history of syphilis. When RPR titers were stratified by stage the difference disappeared in secondary syphilis, in contrast with primary and early latent syphilis where RPR titers were higher in HIV-positive patients. There were no differences between the two groups in treatment received, evolution of RPR or cure of syphilis after 1 year of treatment (*p* = 0.2).

## Discussion

In our study the dominant profile of the epidemic was MSM. Globally in high-income settings, MSM accounts for a disproportionate burden of syphilis infections [19]. This study shows that this cohort of patients with early syphilis, especially HIV-positive patients, engage in high-risk sexual behaviors. At our clinic we had previously found an HIV coinfection rate of 30% in a study conducted between 2003 and 2013 [20]. In this study, we show that this relationship has persisted over time, as we have now found that 36% of patients with syphilis were HIV-positive and 8.5% were newly diagnosed concomitantly with syphilis. HIV and syphilis are related to each other [21, 22], and syphilis itself could increase the risk of HIV infection [23]. However, this increased risk could also be associated with sexual behavior, as HIV-positive patients engage in more high-risk behaviors than HIV-negative patients as we report here, similarly to others researches [24–27]. Factors such as sex in group, multiple sex partners, CAS and use of drugs for sex, could also each raise the risk of syphilis [3, 7]. However, these factors are mutually linked, a fact that might increase the probability of infection. An example of this interrelationship is chemsex, a growing global phenomenon which has been associated with a higher number of partners and CAS, situations in which patients more likely to be diagnosed with acute rectal or bacterial STIs, and HCV [28].

From a clinical perspective, HIV may modify the clinical presentation [22] and course of syphilis. After adjusting for a previous history of syphilis, we found that there were still differences in the stage depending on HIV status, with secondary syphilis being more common among HIV-positive patients, similar to the findings of Hutchinson et al. [9] An atypical presentation of syphilis, such as multiple chancres and concurrent primary chancres in secondary syphilis, had been described more often in HIV-positive patients [8, 22]. In our study we found no such differences with the exception that HIV-positive patients had more anal chancres. This could be explained by seropositioning, in addition to the fact that HIV-positive patients practiced receptive anal sex more often than HIV-negative patients. When taking into account *T. pallidum* typing, molecular studies have associated some strain types with clinical outcomes, such as neurosyphilis [10], ocular involvement [11], or serofast status [12]. Following this methodology, we explored whether or not multiple chancres and other clinical manifestations of early syphilis could be related to a specific strain. We found a high diversity of strains in a limited sample size, precluding any associations. More refined molecular methods such as multilocus sequence typing (MLST) or whole genome sequencing could perhaps be more conclusive [29]. We speculate that HIV could influence the clinical presentation of syphilis in a way similar to how immunological factors affect the manifestation of syphilis in patients with a history of syphilis [30].

Factors that influence the natural course of an infection include host susceptibility, bacterial characteristic and transmissibility, and host immunity [31]. Rekart et al. [32] suggested that ART could increase the susceptibility to syphilis due to changes in the innate and adaptive immune responses to *T. pallidum* by suppressing mitochondrial function, proinflammatory response, and macrophage activation. They concluded that the immunological effects of ART and ART-induced behavioral changes could act synergistically to increase susceptibility to syphilis. Could this be the reason why HIV-positive patients are more likely to have secondary syphilis than HIV-negative patients? In view of our findings on risk behaviors in HIV-positive patients compared with HIV-negative patients, we suggest that sexual behavior probably has a much greater effect than other factors, as Tuddenhem et al. [33] argued.

This study did have several limitations. First, it was carried out at a single center in Barcelona and, therefore, the findings could not be generalized. Nevertheless, the clinic is a known referral center attending a large number of patients with high-risk sexual behaviors and the main findings are in line with those reported by others elsewhere. Another limitation is the use of self-reporting, sometimes with partial information, an approach that could have influenced some of the results. Apart from the inherent constraints of this method, the limited number of samples for molecular typing made it impossible to gather relevant information on the clinical manifestations of syphilis. Further results on microbiological typing in syphilis obtained in this study will be published elsewhere (Fernandez-Naval C. et al, submitted). Last, the sample size is underpowered to make multiple comparisons between groups but the results are consistent with those of other studies.

## Conclusion

In conclusion, HIV-positive MSM patients are more likely than HIV-negative patients to have risk factors for syphilis, such as anonymous contacts, CAS, and chemsex. These factors facilitate syphilis acquisition and and have been found to contribute to the syphilis epidemic in Western countries. HIV-positive patients practicing receptive anal sex and seropositioning may cause them to have anal chancres more often than HIV-negative patients. HIV could influence the natural course of syphilis as secondary syphilis has been found to be more common in HIV-positive patients. These findings are an updated report on syphilis in Barcelona and provide important information to be considered for public health interventions, including STI screening and, access to health services and risk-reduction programs targeting high-risk groups.

## Data Availability

The datasets generated and/or analyzed during the current study are not publicly available due to the sensitivity of the topic and hence to ensure confidentiality of the information but are available from the corresponding author on reasonable request.

## Abbreviations

ART: Antiretroviral therapy
CAS: Condomless anal sex
CI: Confidence interval
CT: *Chlamydia trachomatis*
GHB: Gammahydroxybutrate
HCV: Hepatitis C virus
HIV: Human immunodeficiency virus
IQR: Interquartile range
MSM: Men who have sex with men
NG: *Neisseria gonorrhoeae*
OR: Odds ratio
PCR: Polymerase chain reaction
RPR: Rapid plasma reagin
SD: Standard deviation
STIs: Sexually transmitted infections
